# Impairment on a self-ordered working memory task in patients with early-acquired hippocampal atrophy

**DOI:** 10.1016/j.dcn.2016.06.001

**Published:** 2016-06-03

**Authors:** Sharon Geva, Janine M. Cooper, David G. Gadian, Mortimer Mishkin, Faraneh Vargha-Khadem

**Affiliations:** a Cognitive Neuroscience and Neuropsychiatry Section, University College London Institute of Child Health, 30 Guilford Street, London WC1N 1EH, United Kingdom; b Developmental Imaging and Biophysics Section, University College London Institute of Child Health, 30 Guilford Street, London WC1N 1EH, United Kingdom; c Laboratory of Neuropsychology, National Institute of Mental Health, Bethesda, MD 20892, USA

**Keywords:** aMCI, amnesic mild cognitive impairment, CMS, children memory scale, DLPFC, dorsolateral prefrontal cortex, ECMO, extracorporeal membrane oxygenation, FSIQ, full scale IQ, IFG, inferior frontal gyrus, RT, response time, SOPT, self-ordered pointing task, TGA, transposition of the great arteries, VLPFC, ventrolateral prefrontal cortex, WAIS, Wechsler adult intelligence scale, WISC-IV, Wechsler intelligence scale for children IV, WMS, Wechsler memory scale, Working memory, Hippocampus

## Abstract

One of the features of both adult-onset and developmental forms of amnesia resulting from bilateral medial temporal lobe damage, or even from relatively selective damage to the hippocampus, is the sparing of working memory. Recently, however, a number of studies have reported deficits on working memory tasks in patients with damage to the hippocampus and in macaque monkeys with neonatal hippocampal lesions. These studies suggest that successful performance on working memory tasks with high memory load require the contribution of the hippocampus. Here we compared performance on a working memory task (the Self-ordered Pointing Task), between patients with early onset hippocampal damage and a group of healthy controls. Consistent with the findings in the monkeys with neonatal lesions, we found that the patients were impaired on the task, but only on blocks of trials with intermediate memory load. Importantly, only intermediate to high memory load blocks yielded significant correlations between task performance and hippocampal volume. Additionally, we found no evidence of proactive interference in either group, and no evidence of an effect of time since injury on performance. We discuss the role of the hippocampus and its interactions with the prefrontal cortex in serving working memory.

## Highlights

• Patients with early onset hippocampal damage were impaired on a working memory task.
• Impairment was evident only on those trials when memory load was intermediate.
• Hippocampal volume correlated with behaviour when memory load was intermediate/high.
• Patients showed no proactive interference.
• Patients showed no effect of age at injury on performance.

## 1 Introduction

One of the striking features of both adult- and developmental- onset amnesia produced by damage to the hippocampus is the sparing of working memory (e.g. Milner, 1966, Cave and Squire, 1992). Typically, despite their severe and chronic impairment in episodic or event memory (Allen et al., 2014, Baddeley et al., 2011, Baddeley et al., 2010, Hurley et al., 2011, Vargha-Khadem, 1997), amnesic patients often display normal working memory on such standard tasks as digit span and block span (but see Rose et al., 2012). Given this profile, it might be surmised that the hippocampus makes no contribution to working memory, an ability that is therefore often assumed to be served instead by neocortical regions, especially the prefrontal areas implicated in the maintenance and manipulation of on-line information (Brahmbhatt et al., 2008, Molteni et al., 2008, Vuontela et al., 2009). However, because of their reciprocal neuroanatomical connections (Aggleton et al., 2015, Barbas and Blatt, 1995, Carmichael and Price, 1995, Goldman-Rakic et al., 1984, Kondo et al., 2005, Saleem et al., 2008), severe damage to the hippocampus can potentially compromise the working memory function of the prefrontal cortex.

Heuer and Bachevalier (2011) induced bilateral hippocampal lesions in neonatal monkeys and tested the animals as adults on (a) a self-ordered object-sequence task known to depend on the functional integrity of the dorsolateral prefrontal cortex (DLPFC), and (b) a session-unique delayed non-matching-to-sample task known to depend on the integrity of the ventrolateral prefrontal cortex (VLPFC). Results indicated a selective deficit in the self-ordered working memory task. In contrast, there was no effect of the neonatal hippocampal lesions on the session-unique, delayed non-matching-to-sample task, suggesting that the VLPFC of the hippocampal-lesioned monkeys was functionally intact. In a subsequent report, Heuer and Bachevalier (2013) contrasted the performance of the same monkeys on two different serial order working memory tasks that measure memory for temporal order of stimuli, one being DLPFC- dependent, and the other not. Once again, results confirmed that the operated monkeys were impaired only on the more complex temporal order list—*viz,* the version that is dependent on the functional integrity of the DLPFC. Taking the results of the two studies together, the authors conclude that early hippocampal lesions “…yield significant deficits in…monitoring of information in working memory. The results further suggest that the deficits may relate to an alteration of hippocampal-prefrontal interactions” (Heuer and Bachevalier, 2013, p. 11).

To compare directly the contributions of prefrontal cortex and the medial temporal lobes to performance on the Self-ordered Pointing Task (SOPT), Petrides and Milner (1982) studied groups of adult patients with unilateral surgical excisions of one or the other of these two brain regions. The results confirmed the sensitivity of the SOPT to frontal lobe lesions, especially for removals on the left side, but also indicated that patients with large temporal lobe removals extending medially and posteriorly (i.e. those involving radical excisions of the hippocampus along with parahippocampal gyrus and entorhinal cortex) were impaired as well. These patients showed material-specific deficits on the verbal or nonverbal versions of the SOPT consistent with the side of surgery; i.e. left hemispheric surgery resulted in impaired performance on tasks involving low- and high-imagery words, whereas right hemispheric surgery resulted in impaired performance on tasks involving abstract designs and representational drawings. Relevant to the current study, the report by Petrides and Milner (1982) also highlighted the effects of memory load, with both frontal and medial temporal lobe lesions yielding error rates that increased as a function of increasing number of items in each test block.

The issue of memory load was recently addressed in other reports, where it has been suggested that whether the hippocampus plays a role in working memory depends on both memory load (Axmacher et al., 2010, Axmacher et al., 2007, Jeneson et al., 2012, Jeneson et al., 2011, Jeneson et al., 2010) and memory lag (Elliott and Dolan, 1999, Jeneson et al., 2012, Jeneson et al., 2011, Olson et al., 2006, Owen et al., 1995). Jeneson and Squire (2012) argued that ‘supra-span’ demands (i.e. higher memory loads and longer delays) require long-term memory and, therefore, the participation of the hippocampus, since working memory capacity is overloaded. Others suggest that the hippocampus is involved in the performance of a working memory task when the task requires relational memory, irrespective of whether working- or long-term memory is involved (Hannula et al., 2006, Watson et al., 2013); when information coding and binding occurs (Nee and Jonides, 2013, Nee and Jonides, 2008, Oztekin et al., 2009); when dealing with novel stimuli (Rose et al., 2012); during on-line maintenance of the stimuli for the purpose of active processing (Voss et al., 2011, Warren et al., 2011), or simply during higher order visual spatial processing (reviewed in Cowell et al., 2010, Lee et al., 2012).

We examined the consequences of relatively selective hippocampal damage on performance on the SOPT after early injury in humans. From a cohort with a documented history of hypoxic-ischaemic events early in life, we recruited a large group of patients who showed a moderate to severe degree of hippocampal damage. The timing of the hippocampal lesions, and the time lag between lesion-onset and test in this group of patients resemble the rhesus monkeys studied by Heuer and Bachevalier, 2011, Heuer and Bachevalier, 2013. In the current study we focused on the relation between memory-load and the role of the hippocampus. Building on the growing evidence that degree of hippocampal activation is correlated with memory load (e.g. Jeneson et al., 2012, Jeneson et al., 2011, Jeneson et al., 2010), we attempted to relate the degree of hippocampal atrophy to behavioural performance on the SOPT. This approach contrasts with one that treats hippocampal atrophy as present or absent, as is often the case in patient studies. We adapted the abstract designs version of the SOPT developed by Petrides and Milner (1982), a visual working memory task with varying memory loads, to suit the young age level of our patients and controls. Low memory load trials fall within the span traditionally associated with working memory (Alvarez and Cavanagh, 2004, Cowan, 2001, Luck and Vogel, 1997), whereas high-memory-load trials would potentially exceed working memory span. We hypothesised that the patients’ performance would show a hippocampal-dependent load-effect, i.e., the greater the hippocampal atrophy, the lower the memory load it could maintain. Theoretically, the hippocampal-dependent load effect could also be demonstrated in healthy controls. It is therefore conceivable that at high memory-loads, healthy controls would also find it difficult to hold in mind the order of pointing they generated in each block. Under such circumstances it would be predicted that *both* patients and controls would surpass the limits of their working memory capacity and make increasing number of errors at high memory loads.

Lastly, our patient group is unique inasmuch as the hippocampal damage in each case was acquired in infancy or early childhood. This could have a different effect on function than it would in adults who had developed normally and only later sustained damage to the hippocampus. We therefore also tested the relationship between elapsed time since damage and behavioural performance on the SOPT.

## 2 Materials and methods

### 2.1 Participants

Eighteen patients (age range = 10–33, mean age = 16.7 ± 6.6, 10M, 8F) with confirmed bilateral hippocampal atrophy and Full Scale IQ (FSIQ) within the normal range (FSIQ ≥ 85) participated in the study. Patients had sustained hippocampal damage as a result of a hypoxic- ischaemic event early in life (during infancy or early childhood) due to various aetiologies (acute respiratory failure followed by Extracorporeal Membrane Oxygenation (ECMO) treatment, n = 6; Transposition of the Great Arteries (TGA) and open heart surgery, n = 5; neonatal asphyxia, n = 4; pre-term birth, n = 1; hypoglycaemia, n = 1; epilepsy related, n = 1). Hippocampal atrophy was defined as ≥15% volume reduction on each side (volume reduction averaged across hemispheres: range = 15.7–61.9%, mean = 34.9 ± 15.9%), relative to the mean of a group of healthy controls (n = 64; mean = 3248.64 ± 255.45 mm^3^). Table 1 presents patients’ clinical and demographic information. Eighteen healthy volunteers (age range = 9–38, mean age = 18.1 ± 8.9, 10M, 8F) also participated in the study. Participants had no genetic syndromes, no overt neurological deficits (e.g. hemiplegia), no central visual or auditory impairments, and all were native English speakers. The two groups were matched for gender. Participants completed these tests as part of a larger study. They were assessed over two to three days and were compensated for their time and expenses. The study was approved by the Local Research Ethics Committee and all participants, and/or their parents/guardians, read an information sheet and gave written informed consent before the start of the study.

**Table 1:** Patients’ demographic and clinical information.

Patient	Age	Sex	Hippocampal Volume Reduction (%)	Aetiology	Age at hypoxic event
1	19	F	61.9	Complicated delivery resulting in neonatal asphyxia	Neonate
2	21	M	57.1	Neonatal asphyxia and recurring respiratory illness	Neonate
3	27	M	54.2	Hypoglycaemia	First hypoglycaemic episode at age 9.5
4	12	M	52.6	TGA	Neonate
5	25	M	51.5	Neonatal asphyxia as a result of the umbilical cord wrapped around the neck during delivery	Neonate
6	33	M	50.0	Extreme prematurity and neonatal asphyxia	Neonate
7	14	F	45.0	Pre-term birth and respiratory distress after birth	Neonate
8	23	M	34.9	Epilepsy related	4.5 years
9	11	F	31.5	Pre-term birth resulting in respiratory failure associated with pulmonary hypertension, requiring ECMO treatment	Neonate
10	11	F	27.0	Neonatal aspiration of meconium followed by ECMO treatment	Neonate
11	16	M	23.3	Acute respiratory failure followed by ECMO treatment	Neonate
12	14	F	22.4	Acute respiratory failure followed by ECMO treatment	Neonate
13	10	F	22.3	TGA	Neonate
14	16	M	21.6	TGA	Neonate
15	12	M	20.5	TGA	Neonate
16	14	M	20.3	Acute respiratory failure followed by ECMO treatment	Neonate
17	10	F	16.8	TGA	Neonate
18	12	F	15.7	Acute respiratory failure due to neonatal aspiration of meconium & ECMO treatment	Neonate

### 2.2 Behavioural testing

**Fig. 1:**
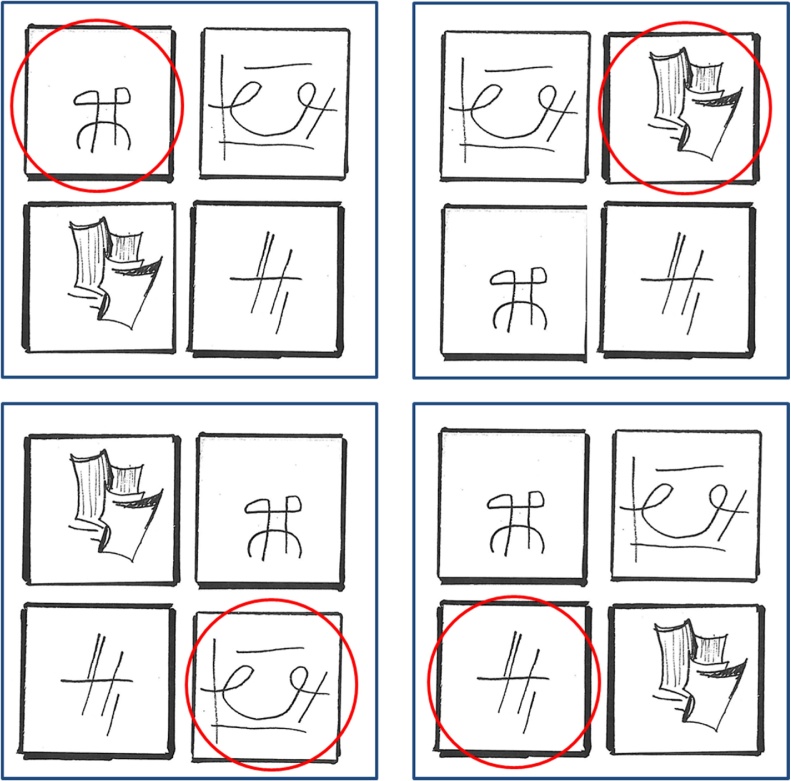
An illustration of a 4- design trial of the SOPT. Each blue square represents a page on which the participant had to point to one design not chosen before. The four designs circled in red provide an example of a possible correct sequence of choices for a 4-designs trial.

For the SOPT, participants were shown an array of abstract designs. The designs were based on those developed by Petrides and Milner (1982), but created anew by a member of our research team, so they are easy to distinguish from one another but difficult to code verbally. Similar to the previous study, the task consisted of different memory load conditions: 6, 8, 10 or 12 designs, with 3 trials in each condition. Here we added a 4-design condition, to ease our younger patients into a gradual increase in memory load. All 3 trials of the same condition were performed consecutively, thus forming a single block. Within each condition, the same designs were used but the location of the designs varied across trials. Hence, in all 3 trials of the 4-designs memory load condition, the same 4 designs were used; similarly, in all 3 trials of the 6-designs memory load condition, the same 6 designs were used, and so on. This allowed testing whether any trials caused proactive interference on subsequent trials, and whether any such proactive interference differentially affected the performance of the two groups of participants. However, each design was repeated only within the same condition, and never across different conditions. The positions of the designs were randomly determined on each page, but the general layout remained the same throughout the task (see Fig. 1).

Participants were shown a grid of different designs and were asked to choose one of the designs and point to it. After making a pointing response, the participant was shown another page, with the same designs appearing in a different order, and asked to point to a different design. Participants were also instructed not to point to the same spatial location more than twice in succession. Therefore, participants were required to remember which designs they had already selected in that trial. This procedure was repeated until participants had the opportunity to choose all the designs once each; thus, in a 4-designs condition, participants were presented with 4 pages; in a 6-designs condition, they were presented with 6 pages, and so on. The order in which the stimuli were pointed to and the time taken to complete the entire trial (i.e. overall response time; RT) were recorded. As in the study by Petrides and Milner (1982), participants were told that accuracy, but not speed, was important in completing the test. It was explained that it was critical to maintain a comfortable pace to proceed through the test (i.e. not so fast that they were unable to examine each item carefully, and not so slow that they would forget which items they had already touched). Reaction times were recorded primarily to ensure that participants were not lingering on specific designs or trials, hence RT was not a primary outcome measure in this study.

In addition, participants completed standardised tests of intelligence (either the Wechsler Intelligence Scale for Children, WISC-IV, or the Wechsler Adult Intelligence Scale, WAIS) and memory (Children’s Memory Scale, CMS, or Wechsler Memory Scale, WMS).

### 2.3 Imaging data acquisition and processing

MRI scans were obtained using a 1.5-T Siemens Avanto scanner, with a T1-weighted 3D FLASH sequence: repetition time: 11 ms, echo time: 4.94 ms, flip angle: 15°, matrix size: 224 × 256, field of view: 250 mm, partition thickness: 1 mm, 176 sagittal partitions in the third dimension, acquisition time: 5.34 min. For the measurement of hippocampal volumes, the datasets were reformatted into 1 mm-thick contiguous slices in a tilted coronal plane perpendicular to the long axis of the hippocampus using MEDx 3.43 (Medical Numerics, Inc., Maryland, USA). Hippocampal cross-sectional areas were measured as described previously (Cooper et al., 2015) by one of the authors (DGG) along the entire length of the hippocampus, using every slice. The volumes were calculated by summing the cross-sectional areas and multiplying by the distance between the measured slices. A correction was made for intracranial volume, and the volumes are presented here in this corrected form. For all participants, measurements were made blind to all clinical data, and to patient or control status.

### 2.4 Statistical analysis

The following variables were analysed: (a) Average number of errors for every memory load condition was compared to chance level. The average number of errors that can be accrued if performance were at chance level is 1.26, 1.99, 2.74, 3.49 and 4.22 errors (based on permutation calculations), on the 4-, 6-, 8-, 10- and 12-design conditions, respectively. (b) Error score: Number of errors in each condition, averaged over the three trials, divided by [number of items-1]. (c) Standardised RTs: RTs averaged over the 3 trials of the same condition, divided by the number of designs in the condition. (d) Incremental RT: the difference in RT between two adjacent conditions. (e) Error rate: Average error rate calculated according to trial order, producing average error rate in the first trials of all memory load conditions (4-, 6-, 8-, 10- and 12-designs conditions); average error rate in the second trials of all memory load conditions; and average error rate in the third trials of all memory load conditions. Hence, error score summarizes performance for all trials of a certain memory load condition whereas error rate summarizes errors by trial order across the entire task. Note that for the calculation of error scores we explicitly controlled for the number of items selected. For error rate, the number of items selected is equal in all three scores, being (4 + 6 + 8 + 10 + 12) = 40 items pointed to overall.

We used independent sample *t*-tests to compare the patient and the healthy control groups; one-sample *t*-tests to compare participants’ performance to calculated chance levels; multivariate tests to examine effects of group and task, and Pearson’s correlation to examine linear correlations between hippocampal volume and performance. Threshold level for significance was set at p < 0.05, unless stated otherwise.

To examine the effect of time since hypoxic event on task performance, we applied the method of curve estimation using the following models: (a) linear—examining the hypothesis that performance improves linearly over time; (b) logarithmic—examining the hypothesis that improved task performance occurs close to the hypoxic event (because early insult accrues greater compensation as a result of neuronal plasticity), or (c) exponential—examining the hypothesis that improved performance occurs at a time distant from the time of the hypoxic event (because of the maturation of frontal lobe function which gradually unfolds with increasing age).

## 3 Results

### 3.1 Behavioural performance

Independent samples *t*-tests confirmed that the two groups did not differ in age (t = 0.12, p = 0.909), FSIQ (t = 0.61, p = 0.55) or in various components of verbal working memory (digit span forward raw score, t = 1.72, p = 0.094; digit span backward raw score, t = 0.72, p = 0.477; digit span standard score, t = 1.45, p = 0.156; and letter-number sequencing, t = 0.19, p = 0.85, all from the WISC-IV/WAIS).

In contrast, patients performed significantly worse than controls on most of the memory measures of the CMS/WMS (visual delayed, verbal immediate, verbal delayed, and general memory score, independent sample *t*-tests, t > 2.5, p < 0.005 for all, significant after Bonferroni correction for multiple comparisons). Table 2 presents the means and the standard deviations relating to the above analyses.

**Table 2:** Groups’ performance on the various neuropsychological tests. *p*-values are from independent sample *t*-tests comparing the two groups. Threshold *p*-value for significant difference following Bonferroni correction for multiple comparisons is p < 0.005.

	Group	Mean	Std. Deviation	p-value for group difference
Full-scale IQ^a^	Controls	110.39	11.653	0.546
	Patients	108.11	10.731	
Digit Span − Standard score^b^	Controls	10.06	2.711	0.156
	Patients	11.56	3.451	
Digit Span – Forward – Raw^b^ score	Controls	9.17	2.618	0.094
	Patients	10.72	2.803	
Digit Span – Backward – Raw score^b^	Controls	7.39	1.754	0.477
	Patients	7.83	1.948	
Visual Immediate − Standard score^c^	Controls	106.53	12.694	0.007
	Patients	90.50	19.470	
Visual Delayed − Standard score^c^	Controls	103.94	11.244	<0.001*
	Patients	82.28	15.988	
Verbal Immediate − Standard score^c^	Controls	101.88	16.363	<0.001*
	Patients	80.33	14.637	
Verbal Delayed − Standard score^c^	Controls	102.65	14.283	<0.001*
	Patients	74.56	22.781	
General Memory − Standard score^c^	Controls	105.94	15.449	<0.001*
	Patients	77.72	19.423	

a Full-scale IQ—from Wechsler Intelligence Scale for Children (WISC-IV) or Wechsler Adult Intelligence Scale (WAIS).

b Standardised score for digit span from Wechsler Intelligence Scale for Children (WISC-IV) or Wechsler Adult Intelligence Scale (WAIS).

c Memory scores from the Children’s Memory Scale (CMS) or Wechsler Memory Scale (WMS) for adults.

* Significant difference between groups.

**Fig. 2:**
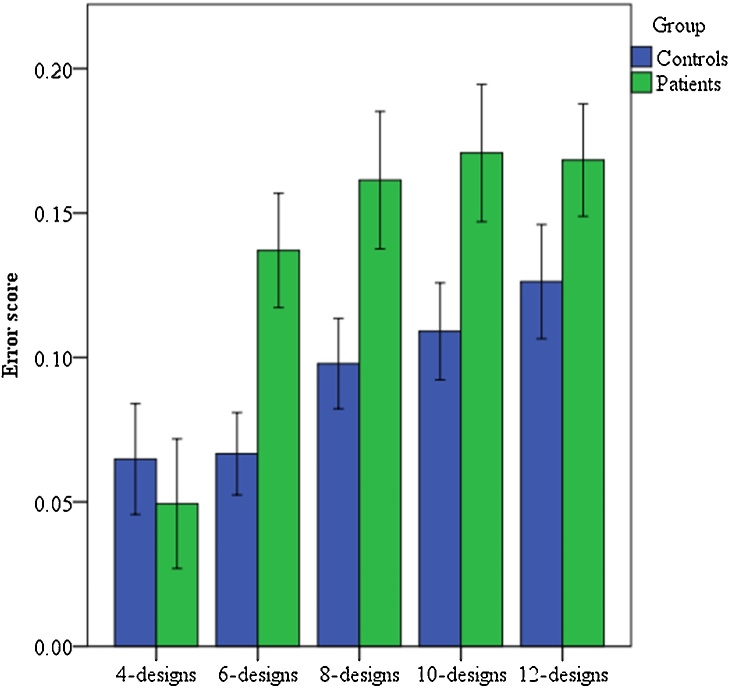
Mean error score in the different conditions (error bars represent ±1 standard error).

Both patient and control groups performed above chance on all conditions (one sample *t*-tests, t > 10, p < 0.001 for all). However, a 5 × 2 multivariate test of the SOPT error score (5 conditions and 2 groups) revealed a main effect of group (F = 5.36, p = 0.027), a main effect of condition (F = 10.55, p < 0.001) and a trend for interaction (F = 2.40, p = 0.067). Conditions also showed a significant linearity effect (F = 34.12, p < 0.001) (see Fig. 2). After controlling for the effects of age and FSIQ by adding those covariates to the model, the group effect remained significant (F = 5.08, p = 0.031), and there was a trend for interaction (F = 2.41, p = 0.067), but the effect of condition was not significant (F = 0.37, p = 0.784). Post-hoc independent samples *t*-tests revealed that the two groups did not differ on the 4-designs condition (t = 0.52, p = 0.604), but they did differ on the 6-, 8- and 10-designs condition (t = 2.88, p = 0.007; t = 2.23, p = 0.032, t = 2.12, p = 0.041, respectively), and did not differ on the 12-designs condition (t = 1.52, p = 0.139) (see Fig. 2).

In summary, although all participants scored significantly above chance, patients performed significantly worse than the control group on the intermediate load conditions (6-, 8- and 10-designs conditions).

Reaction times were not fully recorded for one patient and one control participant. A 5 × 2 multivariate test (5 conditions and 2 groups) comparing standardised RTs revealed a main effect of condition (F = 27.45, p < 0.001), no effect of group (F = 1.93, p = 0.174), and no interaction (F = 3.18, p = 0.084). However, none of the effects were significant after controlling for age and FSIQ (p > 0.05). See Table 3.

**Table 3:** Standardised response times (RTs) for the different conditions in patient and control groups. *p*-values are from independent sample *t*-tests comparing the two groups.

	Controls			Patients			p-value for group difference
	N	Mean	Std. Deviation	N	Mean	Std. Deviation	
4-designs	17	3.13	0.79	17	2.90	1.10	0.50
6-designs	18	3.74	0.99	18	3.54	1.04	0.56
8-designs	18	4.09	1.17	18	3.92	1.25	0.68
10-designs	18	4.98	1.29	18	4.14	1.53	0.86
12-designs	18	5.13	1.22	18	3.89	1.08	0.003*

* Significant difference between groups.

The analysis of the incremental RT examined whether adding two more items when moving from low to intermediate memory load conditions (for example, from 4 to 6 designs), has a different effect on RT than when adding two items at the higher memory load conditions (for example, when moving from 10 to 12 designs). A 4 × 2 multivariate test (4 condition gaps and 2 groups) revealed a main effect of group (F = 8.16, p = 0.007), and task (F = 3.47, p = 0.039) and a significant interaction (F = 4.40, p = 0.044). These effects are driven by a single significant difference (according to post-hoc *t*-tests) in the control group, where the difference in RT between the 8- and 10-designs is significantly higher than all other differences. However, as above, none of the effects remained significant after controlling for age and FSIQ (p > 0.05).

**Fig. 3:**
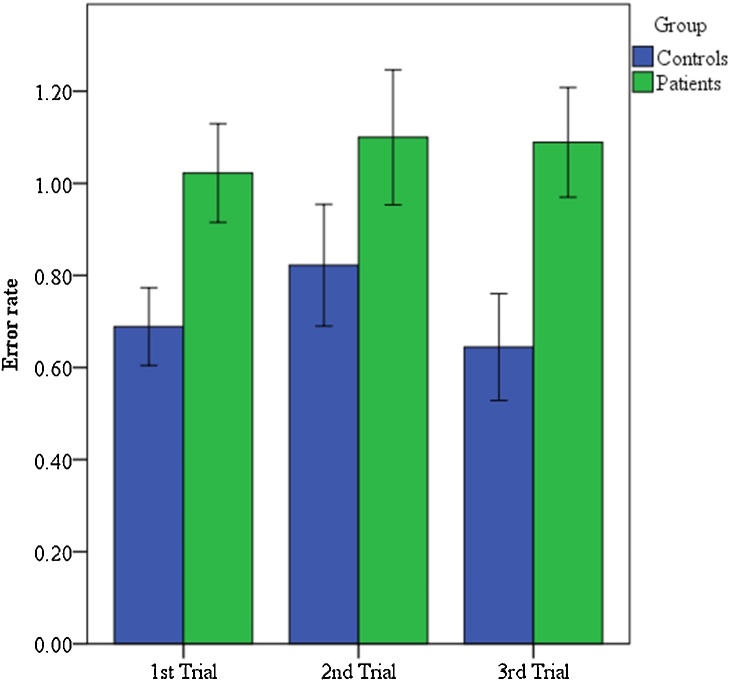
Mean error rate in the 1st, 2nd and 3rd trials, averaged across all memory load conditions (error bars represent ±1 standard error).

Lastly, we examined whether there was evidence for proactive interference within each condition by looking at differences between error rates. A 3 × 2 multivariate test (3 trials and 2 groups) revealed a main effect of group (F = 5.87, p = 0.021), but no effect of trial (F = 1.23, p = 0.3) and no interaction (F = 0.65, p = 0.522; see Fig. 3). We also examined whether there was a significant interaction between error rate and memory load, using a 3 × 5 × 2 multivariate test (3 trials, 5 memory load conditions and 2 groups), but none was found (p > 0.05). These results indicate that participants made a similar number of errors in the first, second, and third trials, thus there was no evidence of proactive interference across trials.

In addition, none of the models; linear, logarithmic or exponential, could significantly explain the patients’ test performance in relation to the time since the hypoxic event (all p > 0.05).

In summary, all participants made more errors in conditions with higher memory load. The control group performed significantly better than the patient group on conditions with intermediate memory load, but the two groups did not differ in reaction time in most conditions and neither group showed evidence of proactive interference from one trial to the next. We also found no effect of time since injury on performance.

### 3.2 Correlations between behavioural performance and hippocampal volume

**Fig. 4:**
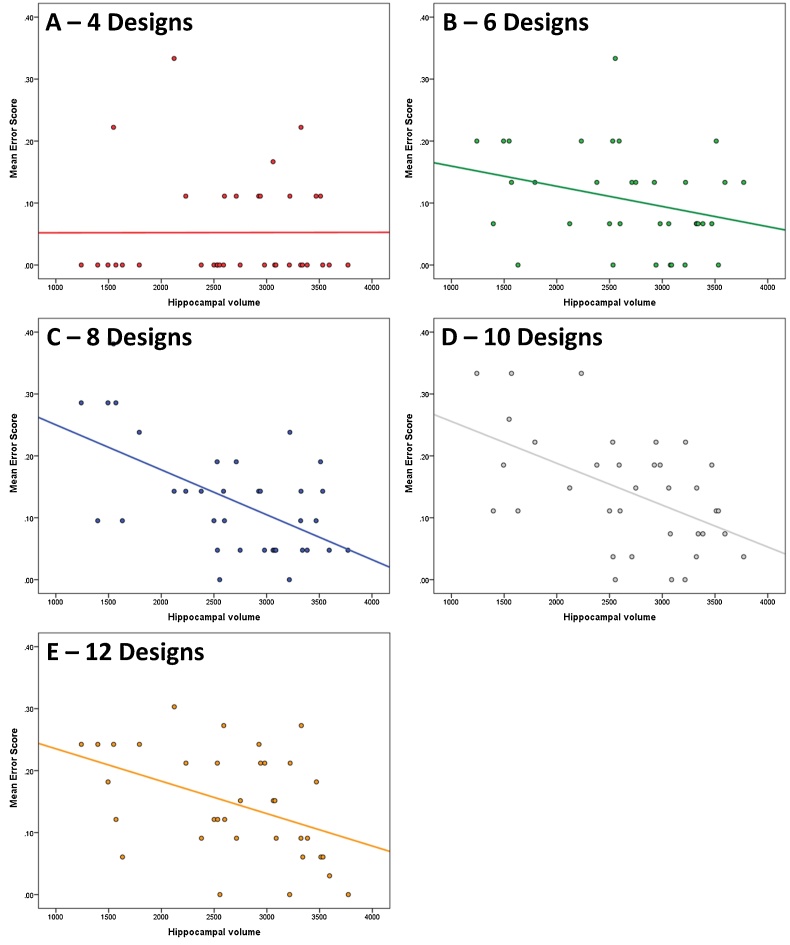
Mean error scores plotted against mean hippocampal volume (mm^3^), for the 4-designs (A), 6-designs (B), 8-designs (C), 10-designs (D) and 12-designs (E) conditions. Lines represent linear regression.

We next examined the relationship between hippocampal volumes and SOPT error scores across all participants. Significant *p*-value for Pearson’s correlation was determined as p = 0.01, after Bonferroni correction for multiple comparisons. There was no significant correlation at the lowest memory load condition (4-designs condition, Pearson’s r = 0.002, p = 0.496), a trend toward significant correlation at the next higher memory load condition (6-designs condition, Pearson’s r = −0.29, p = 0.044), and then significant correlations at each of the still higher memory load conditions (8-, 10-, and 12- designs conditions: Pearson’s r = −0.57, p < 0.001; −0.52, p = 0.001; and −0.43, p = 0.005, respectively; see Fig. 4).

Applying Fisher’s r-to-z transformation followed by a hypothesis test of a point estimate to examine whether these correlations differed significantly from each other, we found that the correlation for the 4-designs condition was significantly lower than those of the 8-, 10- and 12-designs conditions (Z score = 3.09, p = 0.002 for comparisons of 4- and 8-designs conditions; Z score = 2.66, p = 0.007 for comparisons of 4- and 10-designs conditions; Z score = 2.60, p = 0.009 for comparison of 4- and 12-designs conditions). The correlation for the 10-designs condition was significantly higher than that for the 12-designs condition (Z score = 2.81, p = 0.004). No other comparisons were significant (Z < 1.5, p > 0.05).

## 4 Discussion

In this study we found that for intermediate memory loads, performance on a visual memory task requiring self-generated responses was impaired in our patient group with relatively selective hippocampal damage acquired very early in life. These results: (i) expand on similar findings in rhesus monkeys with neonatal lesions (Heuer and Bachevalier, 2013, Heuer and Bachevalier, 2011); (ii) are consistent with deficits on a similar task in adult patients with surgical removals of the medial temporal lobe encroaching on the hippocampus (Petrides and Milner, 1982); and (iii) highlight the importance of the hippocampus in serving working memory performance on tasks with increasing memory load (Jeneson and Squire, 2012). Interestingly, in our patients with a history of neonatal hypoxia-ischaemia leading to hippocampal atrophy, task performance was not influenced by increasing age at test, or by proactive interference. The implications of these several findings will be discussed in turn in the sections that follow.

### 4.1 Effects of neonatal hippocampal lesions in monkeys and humans

There is a striking similarity between the findings of Heuer and Bachevalier, 2011, Heuer and Bachevalier, 2013 in rhesus monkeys with pharmacologically-induced neonatal lesions of the hippocampus tested during young adulthood, and our results in patients with neonatally-acquired bilateral damage to the hippocampus tested during adolescence and young adulthood. In both cases, performance on self-ordered pointing tasks was significantly impaired relative to that of the controls. This consistent pattern of performance across both humans and monkeys raises two important points. First, monitoring of self-selected sequential pointing is disrupted after neonatal/early lesions of the hippocampus, even when these responses are performed online. Furthermore, across both species, the deficit on self-generated sequential pointing is related to increasing memory load (Heuer and Bachevalier, 2013, Jeneson and Squire, 2012). Second, even when bilateral hippocampal lesions are sustained prior to development of any memory or monitoring ability, no other structure can fully compensate for the disruption of this aspect of hippocampal function.

What might be the role of the hippocampus in our task? Various current theories can account for our results. As mentioned in the introduction, some argue that when a task has ‘supra-span’ demands, participants have to draw on information from long-term memory to perform, and therefore the role of the hippocampus is simply to retrieve long- term memories (Jeneson and Squire, 2012). According to Baddeley’s model (2012, Fig. 4), all incoming information is processed by different components of the cognitive system, each of which is, in turn, dependent on long term memory. Depending on the requirements of the information to be processed, working memory draws on one or more components of long term memory to update, modify, or add to incoming information and translate the product to action. This model highlights the interactive nature of working memory with long term memory and its neural substrate, the hippocampus. Others argued that the hippocampus is responsible for the maintenance of the representation during on-line processing (Voss et al., 2011, Warren et al., 2011), even when the task is within working memory limits, as demonstrated before (von Allmen et al., 2013). If the latter is correct then our patients must have used their residual hippocampal tissue to perform even the 4-designs task. Our data do not allow distinguishing between these competing explanations.

### 4.2 Comparison of the effects of unilateral temporal lobectomy versus selective bilateral hippocampal lesions

In adults, large removals of the right hippocampus as part of a temporal lobectomy result in a severe and chronic impairment in the abstract designs version of the SOPT (Petrides and Milner, 1982). Admittedly, the severity of this impairment could be attributable not only to the extent of hippocampal removal, but also to the removal of the posterior parahippocampal gyrus and the anterior medial temporal lobe structures on the right side. These structures provide indirect forward and backward projections to the right dorsolateral prefrontal cortex (Aggleton et al., 2015, Barbas and Blatt, 1995, Carmichael and Price, 1995, Goldman-Rakic et al., 1984, Kondo et al., 2005, Saleem et al., 2008). Furthermore, the removal of the inferior temporal cortices, necessarily included in a temporal lobectomy, could disrupt visual perception itself (Mishkin et al., 1982), thereby potentially contributing additionally to the pronounced deficit on the abstract design version of the SOPT.

### 4.3 Interactions between hippocampus and prefrontal cortex during working memory

Although connections between the hippocampus and the DLPFC are indirect (Aggleton et al., 2015, Goldman-Rakic et al., 1984, Kondo et al., 2005, Saleem et al., 2014), they are implicated in facilitating and maintaining different aspects of DLPFC function, notably the temporal organisation of goal-directed action, a principal component of which is working memory (Fuster and Alexander, 1971, Fuster, 2002). Given this pattern of connectivity, and the late maturation of DLPFC function that stretches to late adolescence and adulthood (Fuster, 2002), it is reasonable to hypothesise that early hippocampal damage can lead to abnormal development of DPLFC function. Hence, although the impairment in our patients’ working memory could be a direct consequence of their hippocampal damage, an alternative or additional explanation must also be considered, viz., that the impairment is the result of a partially disrupted interaction between a directly-compromised hippocampus and an indirectly-compromised DLPFC.

From a cognitive viewpoint, network models of working memory (Fuster, 2009, Petrides, 1994, Ranganath, 2006) have proposed that the hippocampus is responsible for maintaining active representations of novel and complex visual stimuli during short delays, while the prefrontal cortex has a role in selection or inhibition of relevant/irrelevant representations, based on task demands. The 3-state model of short–term memory (Oberauer, 2002) postulates that the medial temporal lobe in general, and the hippocampus in particular, is responsible for information coding and binding; while the ventrolateral prefrontal cortex mediates access to long-term memory. This theory was recently supported by the results of neuroimaging studies using verbal (Nee and Jonides, 2013, Oztekin et al., 2009) and visual (Nee and Jonides, 2008) stimuli. Based on results from an fMRI study, Rissman et al. (2008) suggest that as memory load increases, the ability of the prefrontal working memory system to maintain active representations decreases and this then entails a shift from reliance on frontal structures to reliance on the hippocampus. Voss et al. (2011) have argued that a prefrontal-hippocampal circuit is active during information-seeking behaviour, with the hippocampus being responsible for determining the strength of the on-line memory trace, thereby allowing the frontal lobe to direct behaviour aimed at refreshing this memory trace when needed.

An alternative explanation of our patients’ deficit on the SOPT is the possibility of occult damage to the prefrontal areas, i.e., the damage not being easily detected on conventional structural MRI scans. Indeed, frontal-related behavioural deficits might occur even in the absence of structural damage to the prefrontal cortex, inasmuch as a damaged hippocampus could fail to send normal output to the frontal lobe, creating dynamic frontal diaschisis (Price et al., 2001). However, when such bilateral frontal damage occurs early in life, it is usually diffuse and is likely to affect the development of cognition generally, and speech and language functions specifically, rather than working memory per se (Vargha-Khadem et al., 1985). Together, these considerations lead us to suggest that our patients do not have sufficient frontal lobe damage to explain their load-related working memory deficit.

Clearly, comparing the performance of our patient cohort to that of a group of patients with selective bilateral frontal lobe damage could shed light on the differential contribution of the frontal and hippocampal structures to task performance. However, cases with bilateral frontal damage sustained early in life are extremely rare. Therefore, the question regarding the role of the frontal lobes in high-memory load tasks remains open.

### 4.4 Effect of memory load

On trials where memory load was low (4-designs), performance on the task did not differ between the groups and did not correlate with hippocampal volumes, suggesting that in the current task low memory load does not require recruitment of the hippocampus. However, the lack of correlation should be taken cautiously, since we cannot exclude the possibility that a ceiling effect is at least partially responsible for this result. As memory load increased, however, the patients with hippocampal atrophy performed more poorly than the control group, and this performance *was* correlated with hippocampal volume, suggesting that normal visual working memory with higher memory load requires normal hippocampal function. Note that our patient group was not impaired on standard measures of verbal working memory, which normally appears to be extremely limited (Baddeley, 2012, Cowan, 2008, Fukuda et al., 2010, Marois and Ivanoff, 2005), with many investigators suggesting that it does not exceed four items (Alvarez and Cavanagh, 2004, Cowan, 2001, Luck and Vogel, 1997). Rather, our patients had difficulty only when task demands exceeded the classic definition of working memory capacity, which is traditionally based on span tasks. This impairment was also found in the standardised memory tests, where our patient group was impaired on an immediate memory task (requiring remembering a large amount of information, such as stories). On the most difficult trials (those with 12 designs), the two groups did not differ in their error scores. Therefore, one might argue that the lack of group difference in the highest memory load condition does not support this explanation, and instead, as many have argued before, that the hippocampus is necessary for working memory, long-term memory, or perceptual tasks when the stimuli are sufficiently complex (for reviews, see Cowell et al., 2010, Graham et al., 2010, Lee et al., 2012, Olsen et al., 2012). However, behavioural performance on the highest memory load condition still correlated with hippocampal volume, suggesting that, as we hypothesised, performance on particularly demanding tasks might vary with hippocampal volume even among healthy participants. Together, our findings suggest that a visual working memory task with high memory load, probably exceeding working memory capacity as measured with standard tasks, cannot be performed successfully without the contribution of the hippocampus. Importantly, one can also argue that the hippocampal involvement in the task is a result of both its ability to maintain increasing loads and its importance in managing complex stimuli.

When memory load is kept low and constant, however, other components of working memory, such as maintenance of complex span and speed of processing (Bayliss et al., 2003), can function normally even in the presence of severe bilateral hippocampal damage. Thus, in a series of experiments on patient Jon who has severe hippocampal damage of early onset, cross-modal storage, as well as complex manipulation of visual and verbal stimuli within working memory, were all found to be comparable to those of normal controls (Baddeley et al., 2011).

This complements the results of numerous activation studies in adult humans, suggesting that the hippocampus is involved in working memory in a load-dependent manner (Jeneson et al., 2012, Jeneson et al., 2011, Jeneson et al., 2010). For example, a correlation between increased memory load and increased hippocampal activation was found in an fMRI study of healthy participants (Axmacher et al., 2007) and in intracranial EEG studies of patients with epilepsy (Axmacher et al., 2010, Axmacher et al., 2007). In another fMRI study, activation in the right posterior hippocampus increased as working memory load increased (2-back vs. 1-back condition), however, only when demands on spatial processing were high (Lee and Rudebeck, 2010). Lastly, a relation between an individual’s memory capacity and hippocampal activation within the limits of working memory span has also been documented (von Allmen et al., 2013). Explaining such findings, Jeneson and Squire (2012) argue that ‘supra-span’ demands (higher memory loads/longer delays) necessitate the involvement of long-term memory, and therefore the hippocampus, since working memory capacity is overloaded.

### 4.5 Proactive interference

Interestingly, although the same designs were used within each memory load level, neither group exhibited proactive interference. An early study showed that young rhesus monkeys with bilateral fornix dissection show proactive but not retroactive interference, when associating object and food (Owen and Butler, 1984). Studies of word list learning in patients with amnestic mild cognitive impairment (aMCI), have consistently reported a greater degree of proactive interference in these patients than in healthy adults (Ebert and Anderson, 2009, Hanseeuw et al., 2012, Hanseeuw et al., 2010). Hanseeuw et al. (2012) attributed this impairment to interference either at the encoding or consolidation stage rather than at retrieval. Notwithstanding the many differences between patients with aMCI and our patients, a factor that may have contributed to the absence of proactive interference effects in our sample is the choice of stimuli used, namely, the use of words versus abstract designs. Given that words have a strong semantic component, they are likely to be processed deeply, and so can be more easily retrieved from long-term memory than can nonverbal stimuli. By contrast, the designs used in the current study were both novel and abstract, allowing only superficial levels of processing and therefore more difficult to store in memory. As a result, memory of the designs may well have been too weak to generate any interference. Using more familiar stimuli might have created interference. On the other hand, it might also have eliminated the behavioural deficit documented in this study, as suggested by an early case study (Rose et al., 2012).

### 4.6 Effects of age at injury

We also examined whether time since the hypoxic event could account for some of the variability in patients’ performance. While reorganisation of function can occur following early brain damage (see review by Anderson et al., 2011), it is not clear whether plastic changes and the accompanying behavioural gain occur close to the time of insult, later as a function of interaction with the environment, or continuously throughout maturation and beyond. We tested this hypothesis by examining whether various statistical models can account for the change in performance with time since the hypoxic event. It should be noted, however, that time since the hypoxic event is equal to the patients’ age at test in all but two cases (see Table 1). None of the models yielded significant results, suggesting that elapsed time since the hypoxic event could not account for the variability in performance, supporting previous findings from our group (Vargha-Khadem et al., 2003). While it is likely that our patients experienced some level of structural and functional brain reorganisation, and, as a result, developed behavioural compensatory strategies, these were not sufficient to completely overcome the consequences of bilateral damage to the hippocampus, as verified by the patients' compromised performance. Furthermore, it is possible that both structural and functional reorganisation occur very early in life, but by testing children at later ages, when cognitive functions have emerged, as done here, it is impossible to document the ongoing process itself. Future studies should therefore aim to study very young children to allow for the possible documentation of early plasticity effects, although it must be noted that the behavioural function must first emerge and develop before its trajectory can be tracked during maturation.

## 5 Conclusions

In conclusion, we have demonstrated impairment on a visual working memory task with intermediate memory load, in a group of patients with early onset hippocampal damage. We also found correlations between the degree of hippocampal damage and task performance when task demands were intermediate to high. We suggest that when memory load falls within the traditional definition of working memory capacity the task can be successfully performed even in the presence of severe, bilateral hippocampal damage, but as memory load increases, hippocampal recruitment becomes critical. In the task used here, the transition from working memory independent of the hippocampus to hippocampal-dependent memory occurred beyond 4 items. However, it is unclear whether working memory capacity can be defined based on span tests, since task demands might vary considerably depending on the types of stimuli used, the length of the delays, and other variables related to the specifics of the task (Cowan, 2008, Fukuda et al., 2010). Future studies should aim to specify the contribution of the hippocampus to task performance by manipulating such variables systematically, while disentangling the role of the hippocampus from the putative role of the prefrontal cortex in working memory.

## Disclosures and conflict of interest

The authors have no conflicts of interest with regard to this manuscript.

## Funding

This work was funded by the Medical Research Council (grants Nos. G0300117-65439 and G1002276-98624) and the Intramural Research Program of the National Institute of Mental Health, NIH/DHHS, and supported by the National Institute for Health Research Biomedical Research Centre at Great Ormond Street Hospital for Children NHS Foundation Trust and University College London.

## Acknowledgements

We thank Antonio Incisa della Rocchetta for designing the adapted version of the SOPT, Monica Munoz-Lopez and Eva Zita Patai for helping with recruitment and testing of some of the participants, and Kadharbatcha Saleem for valuable comments on the manuscript.
